# Revolutionary “Clip-Off” Strategy for Macrocycles from Covalent Organic Frameworks

**DOI:** 10.34133/research.0983

**Published:** 2025-11-06

**Authors:** Xiang-Chun Li, Weijie Yang, Wen-Yong Lai

**Affiliations:** State Key Laboratory of Flexible Electronics (LoFE), Institute of Advanced Materials (IAM), School of Chemistry and Life Sciences, Nanjing University of Posts & Telecommunications, Nanjing 210023, China.

## Abstract

The synthesis of complex organic macrocycles, particularly large and rigid variants, has long been hampered by low yields, poor selectivity, and arduous purification stemming from stepwise approaches. Sánchez-Naya et al. present a transformative “clip-off” strategy in *Science*, liberating preformed macrocycles from the pores of designed covalent organic frameworks. By strategically incorporating cleavable bonds into specific covalent organic framework linkers and utilizing precise ozonolysis, near-quantitative yields of macrocycles with ring sizes ranging from 114 to 162 atoms have been achieved. This strategy leverages reticular chemistry to control size and functionality, establishing crystalline frameworks as molecular blueprints.

Organic macrocycles have attracted substantial attention because of their diverse potential applications, particularly in the fields of optics, electronics, chemical sensing, and photothermal conversion [1,2]. The synthesis of large, well-defined organic macrocycles represents a persistent challenge in chemistry [3,4]. Traditional stepwise methods, which involve constructing rings bond by bond from small precursors, encounter increasing complexities as ring size enlarges [5,6]. The stepwise synthesis faces entropic challenges that impede ring closure with larger sizes, while competing oligomerization pathways further diminish the efficiency of macrocycle formation. The entropic penalties impede ring closure, selectivity substantially diminishes, linear oligomers proliferate, and the purification process becomes increasingly arduous [1,7]. In response to these challenges, Sánchez-Naya et al. in a recent *Science* article presented a novel approach: rather than constructing the ring, it is excised from pre-assembled crystalline covalent organic frameworks (COFs) [8,9]. This substantial improvement in scalability utilizes the engineered pores of COFs [10,11]. COFs represent a class of crystalline porous materials characterized by well-defined polygonal skeletons and aligned discrete pores [12,13].

The innovative “clip-off” strategy is executed in 2 distinct phases (Fig. 1). Firstly, COFs are designed and synthesized in which the target macrocycle is inherently encoded within the frameworks. Specifically, COFs with a Kagome topology are utilized, featuring alternating hexagonal and triangular pores. Crucially, the degradable olefins are engineered exclusively within the linkers that define the triangular pores. The hexagonal pores, constructed solely from robust condensation linkages (imine, which are later converted to stable amide or imide bonds), serve as the latent macrocycles. A highly selective cleavage reaction, ozonolysis, is performed, targeting only the pre-positioned cleavable bonds. This precise disconnection releases the intact hexagonal pore structures as discrete, soluble macrocyclic molecules. Furthermore, the choice of reductive work-up using dimethyl sulfide or oxidative work-up utilizing ozone determines the peripheral functionality (aldehyde or carboxylic acid, respectively) on the excised macrocycle. The superiority of the clip-off strategy is quantified by its yields exceeding 90% for macrocycles, surpassing those achieved through stepwise methods. Additionally, this approach allows for precise size control through reticular design and chemoselective functionalization.

**Fig. 1.**
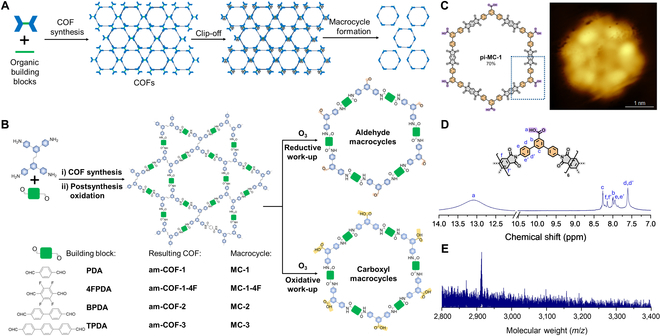
“Clip-off” strategy for macrocycles from covalent organic frameworks (COFs) [8]. (A) Schematic of the approach through excision of organic macrocycles from COFs. (B) Schematic showing that the selective ozonolytic cleavage of olefinic bonds in each isoreticular COF liberates macrocycles. (C) Chemical structure and scanning tunneling microscopy (STM) image of pi-MC-1. (D) ^1^H nuclear magnetic resonance (NMR) spectrum of pi-MC-1. (E) Matrix-assisted laser desorption/ionization-time of flight (MALDI-TOF) spectrum of pi-MC-1.

The effectiveness of the “clip-off” strategy is clearly demonstrated through the synthesis of 9 distinct macrocycles, which illustrates exceptional control over key parameters. The principles of isoreticular chemistry were utilized to expand the COF pores by employing progressively longer linear dialdehyde linkers. This directly facilitated the excision of larger macrocycles: MC-1 (114-atom ring, 35.3 Å in am-COF-1), MC-2 (138-atom ring, 45.2 Å in am-COF-2), and MC-3 (162-atom ring, 51.5 Å in am-COF-3), all of which were obtained in high yields (Fig. 1). The substitution of terephthalaldehyde with tetrafluoroterephthalaldehyde resulted in the formation of a fluorine-decorated COF (am-COF-1-4F), subsequently leading to the synthesis of fluorinated macrocycles. Furthermore, peripheral carboxylic acid groups on the excised macrocycle MC-1-COOH were successfully derivatized with amine-terminated polyethylene glycol chains, a modification that was confirmed by matrix-assisted laser desorption/ionization-time of flight (MALDI-TOF) and nuclear magnetic resonance (NMR) analyses, thereby highlighting the potential for postsynthetic modification.

In addition to the initial polyamide-linked macrocycles derived from amine–aldehyde chemistry, which were subsequently converted to amide structures, a polyimide-linked macrocycle (pi-MC-1) was synthesized (Fig. 1). This synthesis involved the creation of an imine-linked COF utilizing a linker that contained an alkyne. Following this, postsynthetic modification was conducted, wherein imine bonds were exchanged for imide bonds through the introduction of a dianhydride, resulting in the formation of pi-COF-1. The alkyne bonds were then cleaved using ozonolysis, which liberated the rigid polyimide macrocycle. Scanning tunneling microscopy imaging of sublimated pi-MC-1 rings on Au(111) provided direct visual confirmation of their formation and allowed for accurate assessment of their size (Fig. 1). Solution-state NMR (including ^1^H NMR, ^13^C NMR, heteronuclear single quantum coherence, and heteronuclear multiple bond coherence), despite challenges posed by signal broadening in larger systems, was enhanced with synthesized molecular models and diffusion-ordered spectroscopy experiments (Fig. 1). High-resolution mass spectrometry and MALDI-TOF unequivocally confirmed the molecular mass and formula of all macrocycles (Fig. 1). These efforts confirmed the presence of single, discrete species with hydrodynamic diameters consistent with density-functional-theory-optimized structures. Solid-state characterization techniques, including powder x-ray diffraction and Fourier transform infrared spectroscopy, meticulously monitored the synthesis and transformation of the COF precursors. Furthermore, the insolubility of the largest macrocycle was effectively addressed through the application of quantitative deconvolution solid-state NMR. The visualization of pi-MC-1 via scanning tunneling microscopy provided compelling corroborative evidence for its formation and structural integrity.

This work transcends a specific synthetic achievement by establishing a new paradigm for macromolecular synthesis. By conceptualizing crystalline frameworks not merely as functional materials but as preorganized molecular templates, Sánchez-Naya et al. have opened a pathway to the synthesis of complex, monodisperse macrocycles that are often deemed synthetically daunting or impossible using conventional methods. The near-quantitative yields demonstrated scalability up to the gram scale, and precise control over size and periphery represents important advantages. A key aspect of the clip-off strategy is effectively managing solvent–pore interactions during macrocycle release. When the cleavable bonds are broken, the macrocycle must move from its confined state within the COF pore to a fully solvated state in solution. The clip-off strategy becomes particularly favorable for the synthesis of large macrocycles (e.g., >100-atom rings) with rigid backbones, where traditional stepwise cyclization is severely hampered by entropic penalties and kinetic traps, leading to low yields. In contrast, conventional dynamic covalent chemistry or cyclization-based approaches often retain their efficiency for constructing smaller or conformationally flexible rings that can readily adopt cyclization-competent geometries. Furthermore, the incorporation of reticular chemistry principles for design, along with isoreticular expansion for size tuning, underscores the synergy between framework engineering and molecular synthesis. Incorporating theoretical calculations would substantially enhance the mechanistic discussion surrounding the selective cleavage mechanism and the stability of the macrocyclic structure during and after ozonolysis.

While the clip-off strategy represents a breakthrough, its precision depends critically on the structural fidelity of the COF precursor. As linker length increases to form larger macrocycles, challenges such as interlayer slippage, interpenetration, or local disorder can reduce long-range order. Such defects may compromise the uniformity of the cleavable bonds’ chemical environment, potentially leading to incomplete cleavage, unintended scission at robust linkages, or the release of heterogeneous products. The broader applicability of this approach hinges on the development of alternative cleavable linkages compatible with diverse functional groups. Future efforts could explore photo-, redox-, or acid-labile bonds, which may enable the release of macrocycles bearing sensitive moieties such as hydroxyl or amino groups. Diaziridines and disulfide bonds can be also cleaved reductively under mild conditions to release macrocycles. The key design principle is that the cleavage reaction must be highly selective and should take place under conditions that do not compromise the integrity of the macrocycles. While the Kagome topology is a perfect blueprint for planar macrocycles, the synthesis of topologically complex molecules like catenanes or trefoil knots demands frameworks with nonplanar, interpenetrated, or entangled structures. The preservation of crystallinity during postsynthetic modifications is of utmost importance. It has been observed that the transformation of imine linkages to amides or imides can introduce localized strain or disorder within the structure. Extending the “clip-off” strategy to complex architectures like catenanes or sequence-defined oligomers would require targeting specific COF topologies, such as interpenetrated frameworks, where cleaving specific linkers could liberate mechanically interlocked rings, or helical/ordered frameworks to release sequence-defined strands. The success of this approach hinges on the precise spatial preorganization of the target molecule within the COF and the subsequent implementation of a highly selective cleavage reaction at predetermined sites, which together ensure the directional disassembly of the framework to yield the desired intricate structure. Despite these complexities, advancements in reticular chemistry provide essential tools that enhance the potential of the “clip-off” strategy for obtaining new generations of intricate macromolecules directly from crystalline matrices.

The “clip-off” concept, which has been successfully applied to organic macrocycles, indicates important potential for broader applicability. The selection of olefin and alkyne cleavable bonds suggests considerable promise, further enhanced by the extensive possibilities provided by reticular chemistry. This field encompasses diverse topologies, linkers, and functionalities. Consequently, the approach could be extended to liberate a wide range of structurally sophisticated (macro)molecules, including catenanes, complex cages, sequence-defined oligomers, or segments of organic polymers, all of which can be derived directly from their crystalline precursors. This methodology effectively bridges the gap between the precision of framework crystallization and the solution-processability of discrete molecules. For instance, the macrocycle MC-1, precisely characterized within its parent COF and subsequently excised as a soluble molecule, can now be processed for applications requiring monodisperse entities. The precise pore-size control inherent to reticular design translates directly into defined macrocyclic cavities, which are promising for size-selective molecular recognition in sensing or as well-defined catalytic sites. Thus, exciting avenues for the discovery of novel macrocycles with tailored properties for applications in catalysis, optoelectronics, molecular separation, sensing, and supramolecular materials science are opened. The era of synthesizing molecules by liberating them from their crystalline blueprints has indeed arrived.

## References

[1] Zhu H, Chen L, Sun B, Wang M, Li H, Stoddart JF, Huang F. Applications of macrocycle-based solid-state host–guest chemistry. Nat Rev Chem. 2023;7(11):768–782. 37783822 10.1038/s41570-023-00531-9

[2] Ren S, Qiao G-Y, Wu J-R. Supramolecular-macrocycle-based functional organic cocrystals. Chem Soc Rev. 2024;53(20):10312–10334. 39240538 10.1039/d4cs00654b

[3] Jiang Z, Dong R, Evans AM, Biere N, Ebrahim MA, Li S, Anselmetti D, Dichtel WR, Livingston AG. Aligned macrocycle pores in ultrathin films for accurate molecular sieving. Nature. 2022;609(7925):58–64. 36045237 10.1038/s41586-022-05032-1 PMC9433321

[4] Aggarwal AV, Thiessen A, Idelson A, Kalle D, Würsch D, Stangl T, Steiner F, Jester S-S, Vogelsang J, Höger S, et al. Fluctuating exciton localization in giant *π*-conjugated spoked-wheel macrocycles. Nat Chem. 2013;5(11):964–970. 24153376 10.1038/nchem.1758

[5] Feng L, Zhang J, Zhang J, Cao X, Guo Z, Yuan Y, Wang N. Supramolecular organic framework with multidimensional storage spaces for ultrahigh-capacity iodine capture from seawater. Research. 2025;8:0608. 40822122 10.34133/research.0608 PMC12352584

[6] Gagnon C, Godin E, Minozzi C, Sosoe J, Pochet C, Collins SK. Biocatalytic synthesis of planar chiral macrocycles. Science. 2020;367:917–921. 32079773 10.1126/science.aaz7381

[7] Hodge P. Entropically driven ring-opening polymerization of strainless organic macrocycles. Chem Rev. 2014;114(4):2278–2312. 24392672 10.1021/cr400222p

[8] Sánchez-Naya R, Cavalieri JP, Albalad J, Cortés-Martínez A, Wang K, Fuertes-Espinosa C, Parella T, Fiori S, Ribas E, Mugarza A, et al. Excision of organic macrocycles from covalent organic frameworks. Science. 2025;388(6753):1318–1323. 40536988 10.1126/science.adw4126

[9] Wu J, Zhang S, Gu Q, Zhang Q. Recent progress in covalent organic frameworks for flexible electronic devices. FlexMat. 2024;1(2):160–172.

[10] Yin Y, Zhang Y, Zhou X, Gui B, Wang W, Jiang W, Zhang Y-B, Sun J, Wang C. Ultrahigh-surface area covalent organic frameworks for methane adsorption. Science. 2024;386:693–696. 39509500 10.1126/science.adr0936

[11] Mao Q, Zhu Z, Meng J, Wang T. Intelligent flexible memristors for artificial synapses and neuromorphic computing. FlexMat. 2025;2(2):188–203.

[12] Zhou Y, Xie Y, Liu X, Hao M, Chen Z, Yang H, Waterhouse GIN, Ma S, Wang X. Single-molecule traps in covalent organic frameworks for selective capture of C_2_H_2_ from C_2_H_4_-rich gas mixtures. Research. 2024;7:0458. 39188360 10.34133/research.0458 PMC11345538

[13] Li X-C, Sun H, Wang Z, Yang W, Wang Q, Wu C, Chen J, Jiang Q, He L-J, Xue Q, et al. Interface preassembly oriented growth strategy towards flexible crystalline covalent organic framework films for OLEDs. Nat Commun. 2025;16(1):3321. 40199881 10.1038/s41467-025-58534-7 PMC11978764

